# Concept and development of an interactive tool for trial recruitment planning and management

**DOI:** 10.1186/s13063-021-05112-z

**Published:** 2021-03-06

**Authors:** Ruan Spies, Nandi Siegfried, Bronwyn Myers, Sara S. Grobbelaar

**Affiliations:** 1 Department of Industrial Engineering, Stellenbosch University, Joubert Street, Stellenbosch, 7600 South Africa; 2 Alcohol, Tobacco and Other Drug Research Unit, South African Medical Research Council, Francie van Zyl Drive, Tygerberg, Cape Town, 7505 South Africa

## Abstract

**Background:**

Predicting and monitoring recruitment in large, complex trials is essential to ensure appropriate resource management and budgeting. In a novel partnership between clinical trial investigators of the South African Medical Research Council and industrial engineers from the Stellenbosch University Health Systems Engineering and Innovation Hub, we developed a trial recruitment tool (TRT). The objective of the tool is to serve as a computerised decisions-support system to aid the planning and management phases of the trial recruitment process.

**Method:**

The specific requirements of the TRT were determined in several workshops between the partners. A Poisson process simulation model was formulated and incorporated in the TRT to predict the recruitment duration. The assumptions underlying the model were made in consultation with the trial team at the start of the project and were deemed reasonable. Real-world data extracted from a current cluster trial, Project MIND, based in 24 sites in South Africa was used to verify the simulation model and to develop the monitoring component of the TRT.

**Results:**

The TRT comprises a planning and monitoring component. The planning component generates different trial scenarios for predicted trial recruitment duration based on user inputs, e.g. number of sites, initiation delays. The monitoring component uses and analyses the data retrieved from the trial management information system to generate different levels of information, displayed visually on an interactive, user-friendly dashboard. Users can analyse the results at trial or site level, changing input parameters to see the resultant effect on the duration of trial recruitment.

**Conclusion:**

This TRT is an easy-to-use tool that assists in the management of the trial recruitment process. The TRT has potential to expedite improved management of clinical trials by providing the appropriate information needed for the planning and monitoring of the trial recruitment phase. This TRT extends prior tools describing historic recruitment only to using historic data to predict future recruitment. The broader project demonstrates the value of collaboration between clinicians and engineers to optimise their respective skillsets.

**Supplementary Information:**

The online version contains supplementary material available at 10.1186/s13063-021-05112-z.

## Background

Trials, specifically multi-centre trials, require a large investment of money, human resources and time. Effective planning and trial management are key to conducting trials within budget and timelines. Information about trial duration is essential for management decision-making during the planning and conduct stages of a trial. Principal investigators (PI) need to decide whether a trial is feasible based on the time it would take to complete, funding availability and the authorisations required from governing bodies and agreement terms with clinics, staff, community partners and patients [1–6].

The duration of a trial recruitment phase is typically the most difficult to predict prior to the trial, and to manage during the trial, due to the uncertainty and variability of factors that influence recruitment [4, 7–9]. Factors that may impact on trial recruitment include convenience of trial site and duration to participants, incentives and compensation, study staffing and nature of the intervention. Inaccurate estimations of the recruitment duration often result in prolonged periods required to reach the specified sample size. This directly results in increased costs, which often leads to an exceeded budget. Prolonged recruitment may also result in increasing reluctance on the part of stakeholders—clinics, staff or patients—to participate in the trial [4, 10–12].

Should PIs decide to decrease the sample size due to poor recruitment or to terminate the trial early, study power may be threatened if the target sample size is not reached. In extreme cases, the trial may be at risk of a type II error where results are found to be incorrectly statistically non-significant [10–12]. Abandoned studies are a waste of effort and a financial detriment to a research programme and funding agencies, resulting in complete loss of invested capital [11, 13].

There has been increased research interest in the modelling and prediction of trial recruitment duration (TRD) [7, 14–18]. These techniques have the potential to enable informed decision-making during the pre-trial planning and recruitment phases of the trial and ultimately contribute to greater efficiency in trial management [9]. Limited work has been published on how the outputs of the TRD prediction techniques and other information collected during the trial may be utilised to assist or improve decision-making to address typical difficulties in trial management [3]. Although software packages are available that can assist in this decision-making, they tend to be expensive and may be prohibitively so for researchers in low- and middle-income countries. Spending scarce resources on software purchases during the pre-trial planning phase, and to determine trial feasibility, is undesirable when budgets are limited. In the absence of simple to use, open-source recruitment planning software, many investigators from LMICs rely on experience from prior studies to estimate recruitment rates, but this becomes more complex and less accurate when studies involve multiple sites.

In 2016, trial investigators from the South African Medical Research Council (SAMRC) approached the Health System Engineering and Innovation Hub (HSE&IH) from the Department of Industrial Engineering at Stellenbosch University to partner in developing a tool to aid trial recruitment planning and management. The collaboration aimed to utilise the unique expertise from both the clinical research and industrial engineering disciplines to develop a predictive tool which would require minimal additional knowledge to that typically possessed by a clinical trial investigator and would utilise freely available resources to develop, implement and apply.

The purpose of this paper is (1) to describe the design and development of a trial recruitment tool (TRT) to support recruitment planning and monitoring in both single and multi-site clinical trials and (2) to demonstrate the applicability and usability of the tool in a real-world active cluster randomised controlled trial.

## Method

### Project MIND

Project MIND is a three-arm cluster randomised controlled trial (RCT) comparing the effectiveness of two different resourcing approaches to integrating mental health counselling into chronic disease care relative to treatment as usual (TAU) for reducing hazardous alcohol use and depression and improving HIV and diabetes treatment outcomes [19]. In this trial, patients were recruited from 24 primary care clinics in the Western Cape province of South Africa. These facilities were stratified by urban-rural status before being randomly assigned to either treatment-as-usual or one of two intervention conditions. At each site, study assessors screened patients presenting for routine HIV or diabetes treatment for study eligibility. Eligibility criteria included (i) being at least 18 years old, (ii) taking antiretroviral therapy (ART) for HIV or medication for diabetes, (iii) screening positive for hazardous drinking or depression and (iv) providing consent to all study procedures. Patients receiving other mental health treatment or participating in another study were excluded. If eligible patients were interested in study participation, an appointment was made for the enrolment visit. These procedures were followed until 25 unique participants with HIV and 25 unique participants with diabetes were recruited from each site.

After obtaining consent, the participant completed a computer-assisted personal interview about their HIV or diabetes treatment, common mental disorders and contributing psychosocial factors, perceived health status and health service use. Participants also provided blood samples for HIV viral load and HbA1c testing to assess the extent to which their chronic disease was well-controlled. Participants recruited from an intervention site then received the first of three counselling sessions. Participants were given 6 weeks to complete the three-session intervention and an additional 2 weeks to complete the optional fourth session. All participants were tracked for 6 and 12-month post-enrolment assessments in which the baseline interview was read ministered.

Ethics approval for this trial was obtained from the South African Medical Research Council (EC 004–2/2015), the University of Cape Town (089/2015) and Oxford University (OxTREC 2–17). The Western Cape Department of Health also approved this study (WC2016_RP6_9). The trial is registered with the Pan African Clinical Trials Registry (trial registration number: PACTR201610001825403).

### Development of trial recruitment tool

The specific requirements of the tool were determined in several workshops between the clinical investigators from Project MIND and the engineering researchers from the HSE&IH. During the workshops, specific trial recruitment problems were identified, and the type of information required to potentially mitigate these problems was determined. The findings are summarised in Table 1.

**Table 1:** Information requirements to mitigate typical trial recruitment problems

Phase	Problem	Desirable information requirements for decision-making
Planning	Overestimation of the number of potential participants who meet the criteria and would be willing to participate in trial	• No existing information available
	Underestimation of the time it takes recruit the required sample size	• Expected trial and site recruitment duration • Information on how expected trial duration changes when trial parameters are changed
	Not taking variability into account when predicting recruitment duration	
	Overestimation of the recruitment rate	• No existing information available
Recruitment	Latest recruitment information not available to decision makers	• Current recruitment progress for the trial • Expected end-date of trial recruitment • Current recruitment progress for the site • Expected remaining recruitment duration • Information on potential problematic sites • Information on how expected site duration changes when site parameters are changed
	Recruitment information is not presented in a manner that allows necessary decisions to be made	

Since data collection is a core component of any trial, we determined that the information required for recruitment management should be derivable from the data captured by trial field workers during recruitment. A major information requirement for both the recruitment planning and monitoring phases is expected trial recruitment duration.

A large number of studies have proposed and applied various methods and models for predicting TRD, including both analytical and simulation approaches [9, 14–16, 20–23]. Analytical approaches, such as the Poisson-gamma model applied by Anisimov [21], develop analytical expressions to predict the TRD based on statistical models. Simulation approaches, such as the method proposed by Lan et al. [14], provide a dynamic means of predicting TRD which allows more complex, time-dependent factors to be considered.

In this research, a pragmatic simulation approach is selected to predict the TRD such as to demonstrate how dynamic approaches which incorporate existing trial data may be applied in an interactive tool, allowing application of the approach without requiring statistical expertise. The requirements for the model were to (1) reflect the real-world recruitment process, (2) incorporate existing trial data and (3) be implementable within the tool.

The Poisson distribution was chosen to drive the simulation model based on the nature of the recruitment process and its widespread application to prediction [14, 15, 20, 24–26]. The three assumptions required to use the Poisson distribution for the trial recruitment process are verified in Table 2.

**Table 2:** Verification of the Poisson assumptions in the trial recruitment context

Assumption	Trial recruitment context	Assumption holds
Events taking place during one interval do not affect the next	Each recruitment is independent of the next	Yes
Events take place one at a time	Patients are recruited individually, and recruitment data is recorded one at a time	Yes
The average rate of events remains constant	The assumption has been challenged by Williford et al. [27], but numerous other sources support the assumption [26, 28, 29]	Yes

To ensure that the Poisson distribution was representative of the real-world process, trial screening and recruitment data from Project MIND was analysed using multiple chi-squared goodness of fit tests. With the assistance of trial stakeholders, we selected five trial sites that provided the most accurate representation of the typical recruitment rate, i.e. sites that did not experience any external problems during the recruitment period. From the chi-square tests performed on the real-world data (see Supplementary Material), there was not enough evidence available to conclude that the data poorly fits the Poisson distribution (*p* = 0.14; 0.67; 0.62; 0.18; 0.60). The Poisson distribution was, therefore, used to formulate the simulation technique. The following technique was adapted from Carter [24] to meet the identified requirements of the problem and to increase the efficiency in running the simulation:

1. Sample values from Poisson distribution
2. Each sample value represents recruitments at successive time periods
3. Calculate cumulative values and determine which period goal is met
4. Replicate the process to form samples of expected duration
5. Calculate the mean of each sample
6. Calculate the duration confidence interval based on the sample information

A more thorough explanation to the mathematics behind the technique can be found in the Supplementary Material.

Cluster trials typically consist of multiple sites with the recruitment at each site being independent of the others and the rate at which recruitment occurs differing across sites. Sites have different recruitment goals and may start recruitment at different periods. These factors make it impossible to aggregate the information of different sites to predict the overall TRD. Sites are, therefore, simulated individually, based on the site’s recruitment goal and recruitment rate.

**Fig. 1:**
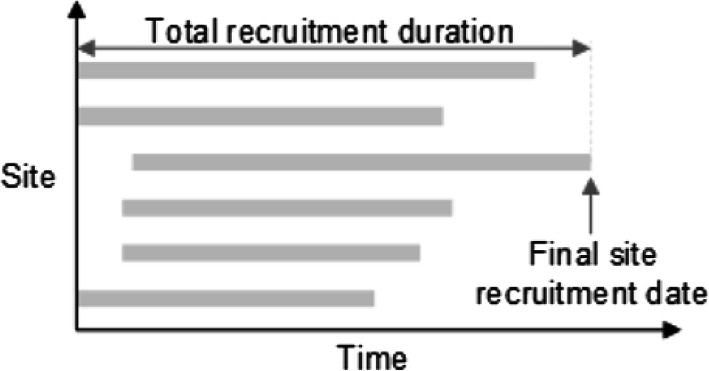
Determining trial recruitment duration by comparing end dates of different sites

The recruitment goal of the site is specified during the trial design phase but may be changed during the recruitment phase, due to various operational factors. For the purposed of the tool, the recruitment rate for each site may be specified a priori or calculated based on the available trial recruitment data. The simulation model is executed at each of the sites, resulting in a predicted TRD for each site. The expected TRD for the trial can then be determined by identifying the latest end date of all the different sites. Figure 1 illustrates this method by depicting the TRD of six different sites.

Due to the resource-constrained environment in which the TRT was to be developed, implemented and used, development on free software was essential. R with the Shiny package is chosen for the development of the *TRT. Shiny* is an R package that allows interactive applications and user-friendly deployable front-ends to be built using R code, a programming language and free software environment [30].

## Results

We developed the TRT consisting of two major components, which are discussed in the following sections. The source code of the TRT, as well as hypothetical sample data which may be used to explore the tool functionality, is available on GitHub and may be accessed at https://github.com/spiesruan/TrialRecruitmentTool.

### Planning component of the TRT

The trial recruitment planning component assists decision-making during the planning phase of a clinical trial and comprises single site and multisite planning.

#### Single site planning

**Fig. 2:**
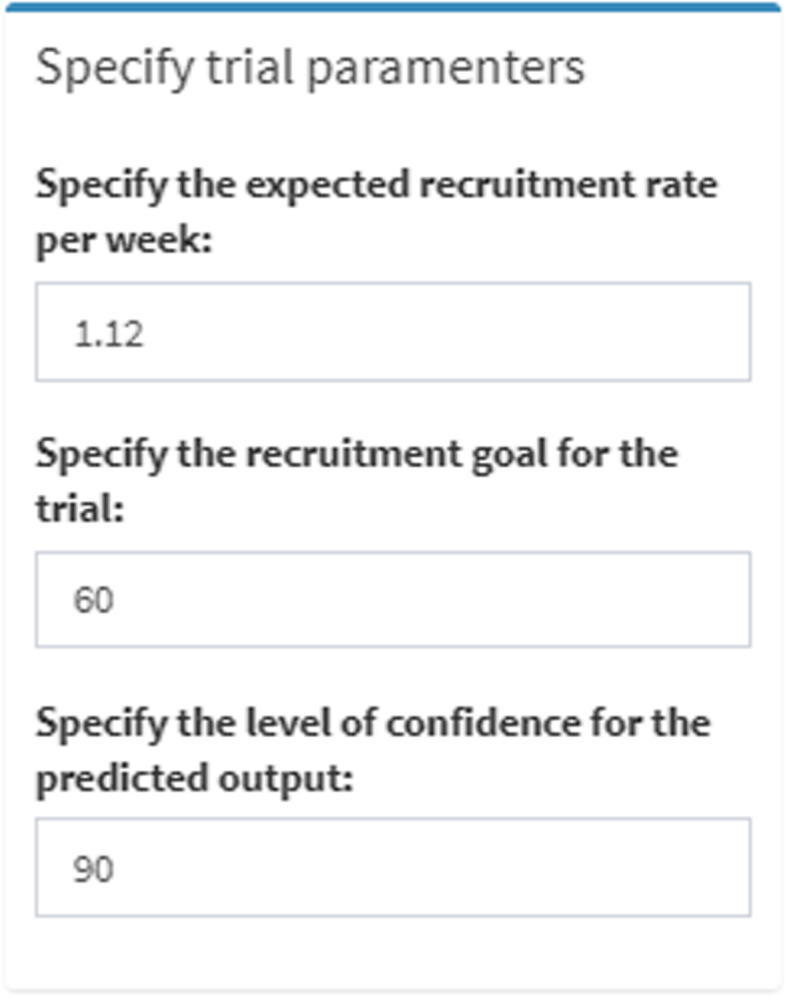
Single site planning user input

The single site planning component provides a rough estimation of how long a trial or site is expected to take to recruit a specified sample size and can be deployed before exact site details are known. Specific known parameters, as shown in Fig. 2, are used by the simulation model to calculate and display the expected TRD. The initial values, which are displayed in the input areas of the TRT, are typical values that would be used for planning based on previous data.

#### Multisite planning

The multisite planning component allows the expected TRD to be predicted with greater accuracy by incorporating several different sites, each with their own parameters. It allows consideration of different scenarios and facilitates planning decisions based on the information presented. Once the number of sites is specified, a table based on the number of sites is generated and the parameters for each site can be inputted. As for single site planning, expected recruitment rate and goals for each site are required, with the option to input initiation delays if the site will only start recruiting after a specified period.

**Fig. 3:**
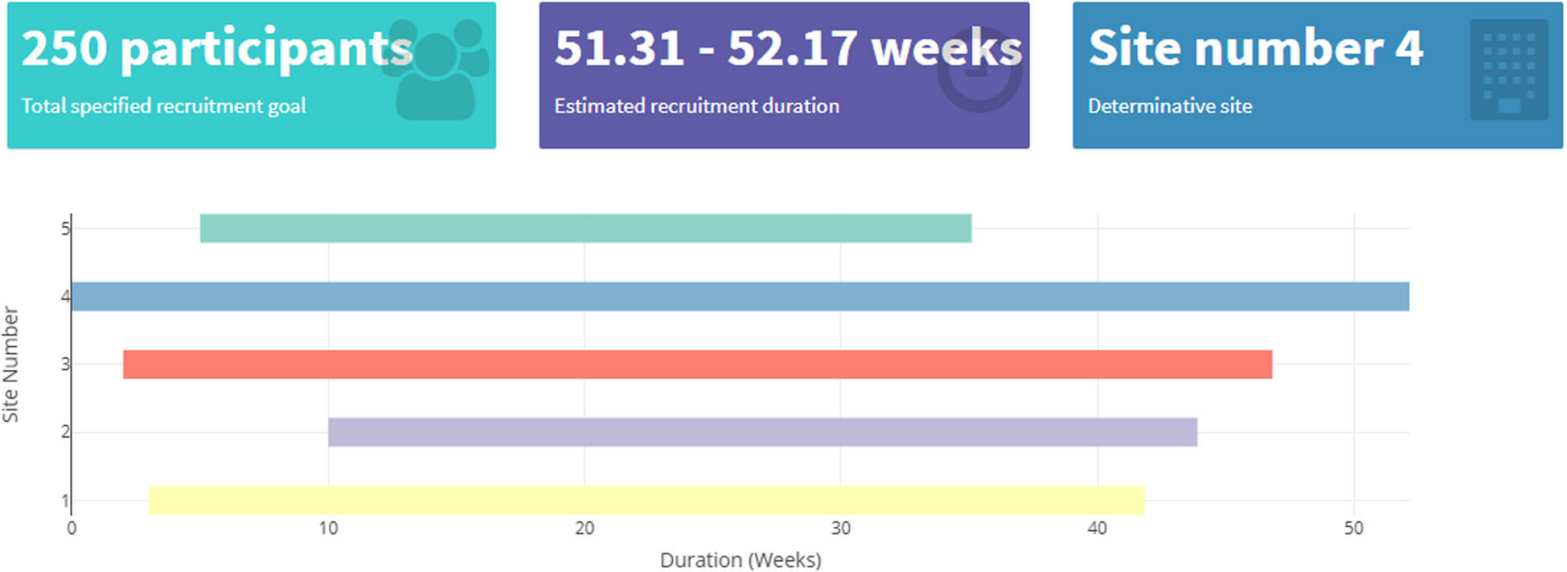
Tool multisite planning output

The input parameters are used to predict the recruitment duration for each site, and the overall TRD displayed on the dashboard is taken to be the duration from the start of recruitment until the last patient is recruited. This may be derived by considering the predicted completion period of the site that is expected to complete recruitment last, termed the determinative site. The results are visualised as shown in Fig. 3.

### Monitoring component of the TRT

The trial recruitment monitoring component uses and analyses the data retrieved from the trial management information system and displays it graphically. Real-world trial data is entered as a comma-separated value file or Excel spreadsheet into the TRT. The data is processed in the background and the three displays, discussed in the following sections, are generated. We demonstrate this application to data from the Project MIND trial.

#### Trial overview component

The trial overview component provides the highest-level summary of the trial progress by displaying a single view of all the current recruitment data. The total number of recruitments and a graph of the cumulative number of recruitments over the entire period are shown. Simulations for each site are performed in the background, based on the provided data and baseline information. The determinant site, with the longest expected remaining duration, is displayed.

#### Site overview component

**Fig. 4:**
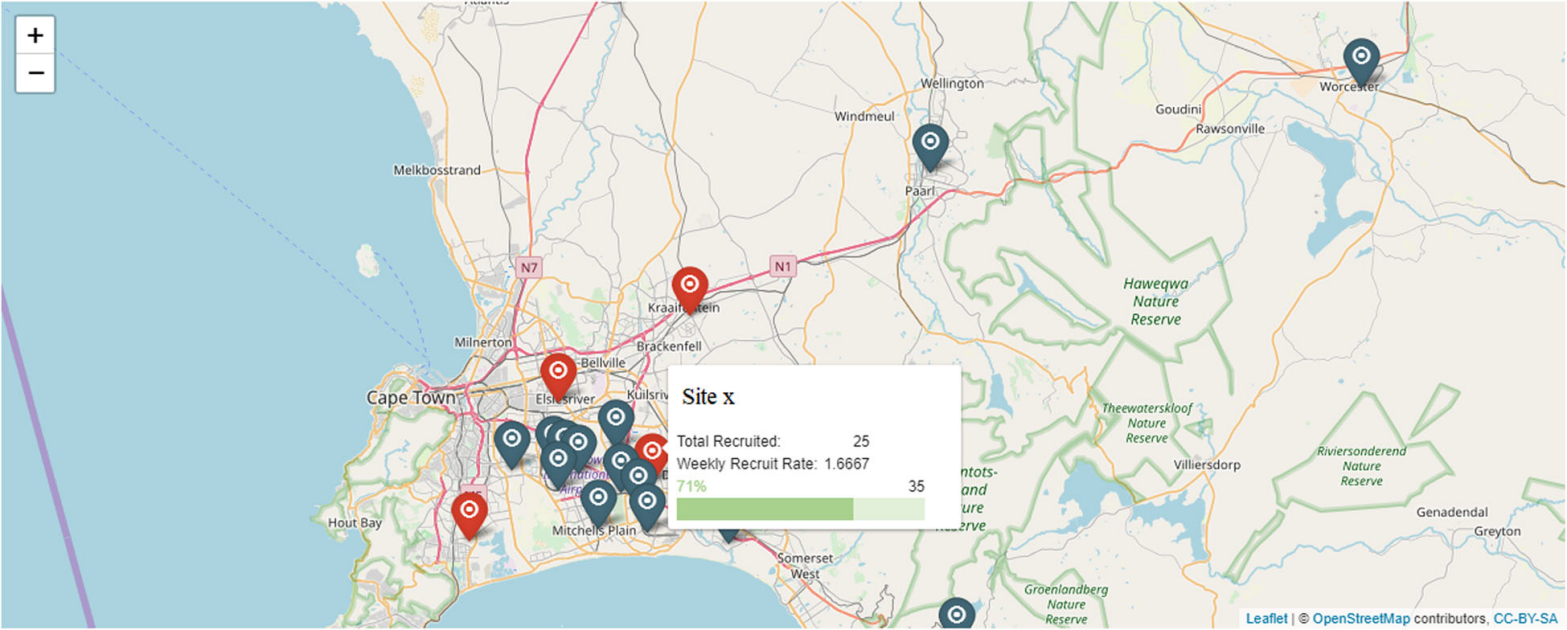
Site overview map display

The site overview component uses the baseline information to plot the different sites on an interactive map. When a site is selected, the site’s recruitment information is displayed in a pop-up information box, as shown in Fig. 4. A specific recruitment quantity can be specified and sites that have less recruitments than specified are shown by red markers.

#### Site specific component

**Fig. 5:**
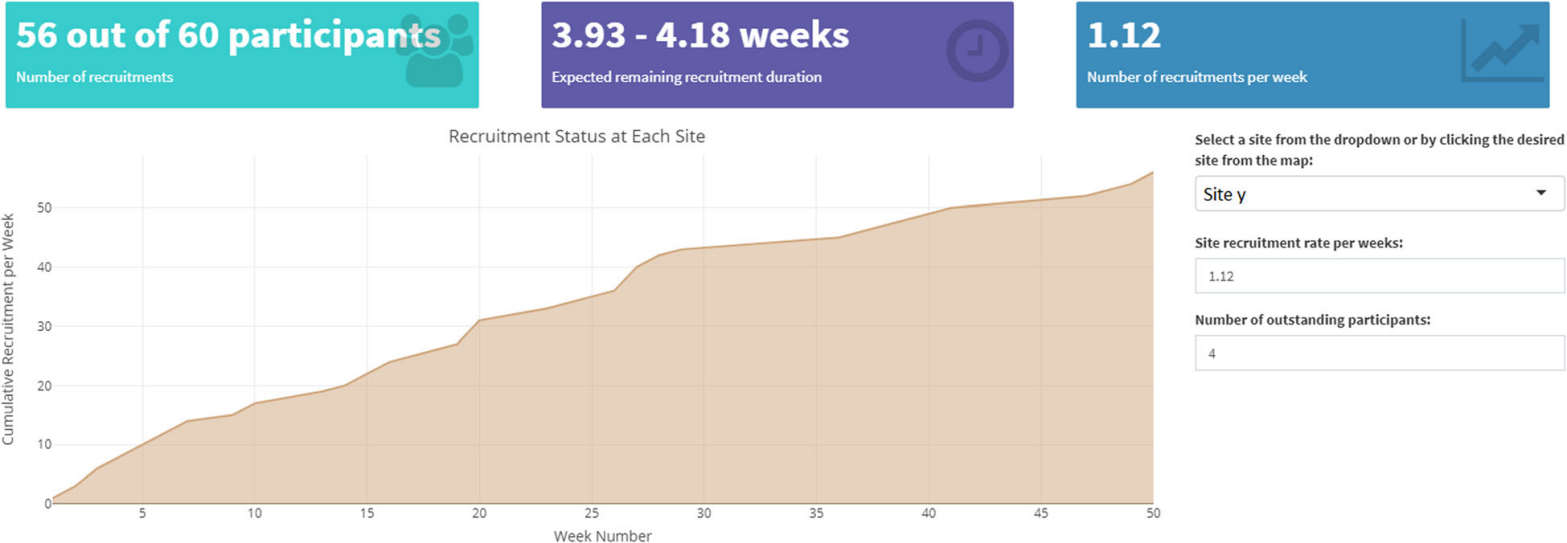
Site specific display

Sites may be further analysed by selecting the site on the map, graph or from a dropdown list. The information of the selected site, as shown in Fig. 5, consists of the actual site recruitment information and the simulated remaining expected duration based on the actual site data. Different scenarios can be displayed by changing the simulation parameters to see the effect of changes in recruitment rate or the number of outstanding recruitments on the predicted remaining duration.

## Discussion

The aim of this study was to develop a tool to support the planning and monitoring of recruitment for clinical trials. The TRT was developed through close collaboration between clinical trial investigators and industrial engineers and has the potential to expedite improved management of clinical trials by providing the appropriate information needed for the planning and monitoring of the trial recruitment phase.

The TRT has multiple uses including (1) enabling evidence-based analysis of the feasibility of conducting a proposed trial within a particular time frame, (2) improved budgeting, resource planning and site selection, (3) provision of important information that can be used for engaging stakeholders and (4) supporting the management and monitoring of the recruitment. These identified aspects are all components of the Clinical Trials Transformation Initiative framework recommended by Huang et al. [4]. Furthermore, the tool is a simple, interactive and responsive tool that requires little resources making it extremely valuable in a resource-constrained environment, as is common in most LMICs.

TRD prediction methods and models have been a growing research domain [9, 14–16, 20–23], but minimal work has been published on how these models may be embedded in the actual trial management context and require specialised knowledge to use and interpret. This study demonstrated how a tool can act as a bridge between theoretical models and the specific information required by a trial manager to make management decisions in the trial context. Alternative and more complex simulation models as well as analytical approaches for predicting TRD may be incorporated in the tool. This would however require potential adaptation of (1) the inputs requested from the user interface, (2) the backend processing of the user inputs and trial data and (3) the model outputs such that it is compatible with all of the tool components.

This study differs from studies that have developed dashboards to provide visual summaries of trial information. Unlike Toddenroth [31] and Mattingly [32], the TRT developed in this study simplifies the trial data and displays only the information required for the actual management of the trial recruitment process, removing redundant data. The dashboards developed in these former studies present descriptive information of historic data whereas the TRT extends this by using the historic data to present predictive information. This is particularly valuable for trial managers who can use this data to be proactive in preventing potential delays in trial recruitment by taking various informed actions, such as allocating additional resources to an underperforming site.

Several limitations of this study need to be recognised. A simple simulation model was formulated to predict the TRD since the aim was to develop a usable and pragmatic solution for demonstration purposes. The TRD predicted by the simulation model is dependent on the accuracy of the specified recruitment rate. During the planning stages, this requires prior knowledge and does not prevent over- or under-estimation of the rate. During the recruitment phase, the recruitment rate is calculated based on the assumption that the rate has been, and will remain, constant over time. This assumption does however not hold in certain cases, such as a site being shut down for a period. The accurate prediction of the recruitment rate is also found to be problematic in practice, particularly at the initial stages when no trial data is available. Furthermore, although our suggested simulation approach appropriately serves the objective of this study, similar results may be predicted from a simpler analytical expression derived from the Poisson process since our approach only considers the basic parameters of the Poisson distribution.

Future research may focus on incorporating more complex TRD prediction approaches in the tool, such as that suggested by Minois et al. [22], to mitigate the former discussed limitations and improve on the assumptions made in this study. The broader study demonstrates the value of collaboration between clinicians and engineers to optimise their respective skillsets for the development of practical and impactful solutions to methodological and trial management challenges. Opportunity exists for further intersectoral collaborations in research and solution development within the clinical trial environment.

## Supplementary Information

**Additional file 1.**

## Data Availability

The tool source code and sample data to demonstrate the tool usage is available in the GitHub repository, https://github.com/spiesruan/TrialRecruitmentTool.

## Abbreviations

ART: Antiretroviral
LMIC: Low- and middle-income countries
PI: Principal investigator
TAU: Treatment as usual
TRD: Trial recruitment duration
TRT: Trial recruitment tool
