# Supplementary material for: Concept and development of an interactive tool for trial recruitment planning and management
Source: Trials. 2021 Mar 6;22:189. doi: 10.1186/s13063-021-05112-z (PMC7936448; doi:10.1186/s13063-021-05112-z)
Supplement: Supplementary file 1 — Additional file 1. [file 13063_2021_5112_MOESM1_ESM.zip › Revised_Supplementary Material A_GOF TestsR2.docx]

Goodness of Fit Tests

The simulation technique uses a statistical distribution to sample random values in order to predict the recruitment duration. It is crucial that the distribution used represents the real-world process. The literature discussed in the article advocates the use of a Poisson distribution to represent the recruitment process. The aim of this section is to validate whether the theoretical Poisson distribution adequately fits the real-world recruitment data.

Recruitment data was obtained from the project up until 22 June 2018. The data posed limitations as it contained a small number of data points. A small amount of data points creates bias in the test, as it decreases the likelihood of the test rejecting the null hypothesis. Despite the potential bias, the tests should still indicate whether the Poisson distribution definitely does not fit the real-world process data.

Project MIND consists of twenty-four sites at which interventions are conducted. The recruitment at each site is independent of the others and the rate at which it occurs differs across sites. Furthermore, sites have different recruitment goals and may start recruitment at different periods. Sites are, therefore, considered individually when assessing the goodness of fit of the theoretical distribution to the data.

With the assistance of trial stakeholders, five sites are selected from the trial that provide the most accurate representation of the typical recruitment rate, i.e., sites that did not experience any external problems during the recruitment period.

#### Visual Inspection

The distribution parameter for each of the sites are calculated from the existing data, and used to determine the expected frequency for the number of recruitments per week. The calculated observed values determined from the real-world data are plotted against the actual values and visually inspected to determine whether it is expected for the theoretical distribution to fit the data.

The plot for Site 1, seen in Figure 1, reflects the scenario where the theoretical distribution is expected to adequately fit the data. Each of the five sites considered, rendered similar results - the four other sites evaluated can be seen in following sections.

Figure 1 Comparison of the frequency in weekly recruitment between real-world observations and theoretically calculated values for Site 1

The visual inspection process serves as an initial indication of what can be expected when conducting a quantitative test. The following section discusses the application of the chi-square GOF test to each of the five sites.

#### Goodness of Fit Tests

The chi-square GOF test is applied to each of the sites to determine whether enough evidence is available to reject the null hypothesis:

$H_{0}:$ $Y$ is Poisson distributed with parameter $\lambda$

Where $Y$is the random variable indicating the number of recruitments per week and $\lambda$ is the Poisson distribution parameter. The results from one of the sites, Site 1, are shown in Table 1.

Table 1 Goodness of fit test for recruitment data for Site 1

| Number of Recruitments | Actual Frequency ($O_{i}$) | Expected Frequency ($E_{i}$) | $E_{i}$' | $O_{i}$' | $\frac{\left( E_{i}-O_{i} \right)^{2}}{E_{i}}$ |
| --- | --- | --- | --- | --- | --- |
| 0 | 10 | 8.2589 | 8.2589 | 10 | 0.3670 |
| 1 | 12 | 12.1590 | 12.1590 | 12 | 0.0021 |
| 2 | 7 | 8.9504 | 8.9504 | 7 | 0.4250 |
| 3 | 4 | 4.3923 | 6.6263 | 7 | 0.0211 |
| 4 | 2 | 1.6166 |  |  |  |
| 5 | 0 | 0.4760 |  |  |  |
| 6 | 0 | 0.1168 |  |  |  |
| 7 | 1 | 0.0246 |  | k = | 2 |
| 100 | 0 | 0.0000 |  | $\chi$^2^_calc_ = | 0.8152 |
| Sample Size | 36 |  |  | $\chi$ ^2^_crit_ = | 5.9915 |
| $\lambda$ | 1.4722 |  |  | p-value | 0.6652 |
| Do not reject $H_{0}$ since $\chi$^2^_calc_ < $\chi$ ^2^_crit_ and p-value > $\alpha$ | | | | | |

The results from the five chi-square tests are summarised in Table 2.

Table 2 Chi-square test results summary

| Trial Site | $\chi$^2^_calc_ | $\chi$ ^2^_crit_ | p-value | Conclusion |
| --- | --- | --- | --- | --- |
| Site 1 | 0.8152 | 5.9915 | 0.6652 | Do not reject $H_{0}$ |
| Site 2 | 2.1754 | 3.8415 | 0.1402 | Do not reject $H_{0}$ |
| Site 3 | 0.2417 | 3.8415 | 0.6230 | Do not reject $H_{0}$ |
| Site 4 | 3.4185 | 5.9915 | 0.1810 | Do not reject $H_{0}$ |
| Site 5 | 1.0155 | 5.9915 | 0.6019 | Do not reject $H_{0}$ |
