# Supplementary material for: Concept and development of an interactive tool for trial recruitment planning and management
Source: Trials. 2021 Mar 6;22:189. doi: 10.1186/s13063-021-05112-z (PMC7936448; doi:10.1186/s13063-021-05112-z)
Supplement: Supplementary file 1 — Additional file 1. [file 13063_2021_5112_MOESM1_ESM.zip › Supplementary Material B_Simulation modelR2.docx]

Simulation model

A total of $w_{max}$ values are sampled from the distribution $Pois\sim(\lambda)$, where $\lambda$ is the distribution parameter, specified prior to the sampling. Each of the sampled values, $y_{w}$, is representative of the number of recruitments that took place during time period $w$, where $w=1, 2, \ldots, w_{max}$.For each period, $w$, the cumulative total of recruitments, up to period $Y_{w}$, is calculated.

The decision maker specifies $N$, the recruitment goal, which is compared to the values $Y_{1}$ to $Y_{max}$. The first period in which the cumulative recruitment value satisfies the recruitment goal, is the simulated prediction of the recruitment duration, denoted by $P_{rs}$. The simulation is repeated $r_{max}\times k_{max}$ times to create samples, $k=1, 2, \ldots, k_{max}$, each containing observations, $r=1, 2, \ldots, r_{max}$. This may then be expressed as

|  | $P_{rs}$ = $\left. \min\left( w \right) \right\vert Y_{w}\geq N$ | For $r\in1, 2, \ldots, r_{max}$ and  $k\in1, 2, \ldots, k_{max}$ |
| --- | --- | --- |

The simulated data is used to make a prediction on the trial recruitment duration by calculating the CI. The CI may be calculated by:

1. Calculate the mean of each sample by computing

|  | $\bar{x}_{k}= \frac{\sum_{r=1}^{r_{max}} P_{r,s}}{r_{max}}$ |
| --- | --- |

for all $k\in1,2, \ldots, k_{max}$.

1. Calculate the mean of each of the means by computing

|  | $x= \frac{\sum_{k=1}^{k_{max}} \bar{x}_{k}}{k_{max}}$ |
| --- | --- |

1. Calculate the unbiased estimator of the population variance by computing

|  | $S_{\bar{X}}^{2}=\frac{\sum_{k=1}^{k_{max}} \left( \bar{x}_{k}-x \right)^{2}}{k_{max}-1}$ |
| --- | --- |

1. Finally, the CI may be calculated as

|  | $CI= X\pm t_{n-1, 1-\frac{\alpha}{2}} \sqrt{\left( \frac{S_{\bar{X}}^{2}}{k_{max}} \right)}$ |
| --- | --- |

where the CI may be interpreted as:

*The decision maker can be* $\left( 1-\alpha\right)\times100\%$ *sure that the true mean recruitment duration falls within the specified interval given that accuracy of the specified recruitment rate.*

The various formulas used in this section rely on both the specified and calculated variables. The specified variables ultimately determine the confidence interval and the width thereof.

The TRT developed in the project may allow users to specify all the required variables to predict the recruitment duration. However, DMs are primarily concerned with the final prediction and want easy access to the information. The TRT design, therefore, aims to eliminate any unnecessary input.

The different variables used by the simulation model are summarised in Table 1, which indicates what will be specified by the DM and what are already built into the TRT. The values specified were chosen by the author based on experience. The values are tested in a hypothetical situation and verified that the output is acceptable.

Table 1 Variables used by the simulation model

| Decision Maker Variables | | Predetermined Variables | | |
| --- | --- | --- | --- | --- |
|  |  | Variable | | Value |
| $\lambda$ | Average recruitment rate | $w_{max}$ | Number of values sampled | 10000 |
| $N$ | Recruitment goal | $r_{max}$ | Number of observations per sample | 10 |
| $1-\alpha$ | Level of confidence | $k_{max}$ | Number of samples | 100 |
