# Supplementary figures and images for: Concept and development of an interactive tool for trial recruitment planning and management
Source: Trials. 2021 Mar 6;22:189. doi: 10.1186/s13063-021-05112-z (PMC7936448; doi:10.1186/s13063-021-05112-z)

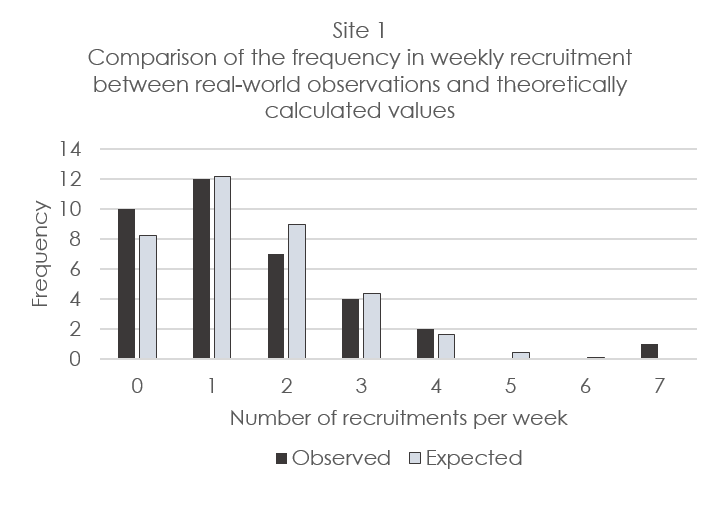

Supplement: Supplementary file 1 — Additional file 1. [file 13063_2021_5112_MOESM1_ESM.zip › Supplementary Material A_Figure 1R2.png]
